# Mechanical Stress Triggers Premature Senescence in Cardiac Fibroblasts

**DOI:** 10.1002/advs.202513314

**Published:** 2025-09-26

**Authors:** Stephanie E. Schneider, Adrienne K. Scott, Katie M. Gallagher, Emily Y. Miller, Soham Ghosh, Corey P. Neu

**Affiliations:** ^1^ Paul M. Rady Department of Mechanical Engineering University of Colorado Boulder Boulder CO 80309 USA; ^2^ Biomedical Engineering Program University of Colorado Boulder Boulder CO 80309 USA; ^3^ Department of Mechanical Engineering Colorado State University Ft. Collins CO 80523 USA; ^4^ School of Biomedical Engineering Colorado State University Ft. Collins CO 80523 USA; ^5^ Translational Medicine Institute Colorado State University Ft. Collins CO 80523 USA

**Keywords:** cellular senescence, mechanical stress, mechanosensitivity, nuclear integrity

## Abstract

The cardiovascular system functions under continuous cyclic mechanical stretch, with disruptions in mechanical and biochemical signals contributing to disease progression. In cardiovascular disorders, these disruptions activate cardiac fibroblasts (CFs) and promote cellular senescence, yet it remains unclear whether mechanical stimuli alone can initiate this phenotype. Here, primary murine CFs are exposed to uniaxial stretch, and systematically varied mechanical parameters assessed their role in senescence induction. Loss of stretch magnitude and increase in frequency, mimicking a pathologic hypertrophy and fibrosis, led to a senescence phenotype, identified through cell cycle arrest, decreased lamin B expression, and DNA damage. Mechanically‐induced CF senescence depends on p53/p21, whereas senescence triggered by oxidative stress or lamin A/C mutation proceeded via p16. Notably, mechanically‐induced premature senescence is accompanied by reduced levels of the nuclear envelope protein emerin. These findings demonstrate that altered mechanical signals are sufficient to trigger premature senescence and implicate compromised nuclear integrity in the underlying mechanism.

## Graphical abstract

Cellular senescence contributes to disease burden in cardiovascular disease (CVD) and aging, highlighting the need to understand its induction. In primary cardiac fibroblasts, reduced strain and increased frequency, mimicking CVD, elicit a distinct senescent phenotype compared to oxidative stress. These findings demonstrate that perturbed mechanical loading alone can initiate senescence.

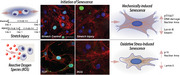

## 1 Introduction

Cellular senescence is a state of cell cycle arrest that plays a role in tissue formation and remodeling during development and following injury.^[^ ^1^, ^2^ ^]^ Senescence can lead to reduced tissue regeneration and function, which contributes to inflammation and promotes tumorigenesis in aging organisms. While traditionally characterized as an arrest in cell cycle, the hallmarks of senescence have been more broadly defined, which include DNA damage, secretory‐associated senescent phenotype (SASP), and apoptosis resistance.^[^ ^3^, ^4^, ^5^ ^]^ Extrinsic factors such as oncogene disruption, irradiation, chemotherapy, developmental cues, and tissue damage have been shown to generate different forms of stress‐induced senescence.^[^ ^1^ ^]^ To further complicate the identification of a senescent phenotype, no one single biomarker is specific to every induction of senescent pathways.^[^ ^6^ ^]^ Instead, multiple biochemical signals, morphological features, and alterations in protein and gene expression are used to define the senescent phenotype.

The most widely recognized markers for defining senescence are through the cell cycle arrest pathways, specifically, p16/p19 in mice (or p16/p14 in humans) and p53/p21.^[^ ^4^, ^7^ ^]^ The p53/p21 axis is also activated with the DNA damage response signaling pathway which can give rise to the senescent phenotype. Biophysical features such as the enlargement of the cell and nuclear area, and increased granularity within the cell occur in addition to biochemical signaling and gene expression changes. Interestingly, many of the hallmarks characterizing the senescent phenotype overlap with biophysical alterations associated with the nucleus such as global changes in histone modifications and chromatin remodeling, and changes at the nuclear envelope such as the loss of lamin B.^[^ ^8^, ^9^ ^]^ Finally, increased excretion of inflammatory cytokines, chemokines, and matrix metalloproteinases generates a persistent feedback loop which prevents a return to homeostasis and instead directs the cell toward apoptosis or senescence.^[^ ^2^ ^]^ Due to the numerous pathways, heterogeneous senescent phenotypes, and the discovery of new initiators of senescence, the characterization of this cellular state needs to be defined in a tissue and disease‐dependent manner.^[^ ^10^ ^]^

Emerging evidence has shown altered mechanical stimulus and microenvironments could provide another factor to initiate a premature senescent‐like state.^[^ ^11^, ^12^ ^]^ With prolonged exposure to physical stresses or stretch deformation as observed in trauma and disease, the cell and nucleus undergo direct changes that influence an array of biomarkers.^[^ ^13^, ^14^, ^15^, ^16^ ^]^ However, if the cellular stress continues outside of a normative physiological range,^[^ ^17^ ^]^ a premature stress‐induced senescent phenotype can emerge. This phenotype is linked with disease states such as cancer, osteoarthritis, and cardiovascular disease.^[^ ^3^, ^18^ ^]^ Notably, these disease states often lead to changes in both biochemical and mechanical environments in the tissue indicating senescence may initiate from a combination of factors.

Due to the many disorders associated with cardiovascular diseases such as hypertension, cardiomyopathy, heart failure, and cardiac hypertrophy, this disease state has become the leading cause of death worldwide.^[^ ^19^, ^20^ ^]^ One common symptom of cardiovascular disease is fibrosis which arises from excessive deposition of extracellular matrix proteins.^[^ ^21^, ^22^ ^]^ The stiffer fibrotic tissue affects cellular contractions in a complex manner which in turn leads to decreased tissue stretch and increased frequency of contraction, and therefore, directly impacts the nucleus.^[^ ^15^, ^23^, ^24^, ^25^ ^]^ Cardiac fibroblasts (CFs) are one of the main cell types involved in the adaptive response to environmental cues in heart tissue. CFs regulate the remodeling and repair process, and the balance between healthy or fibrotic tissue.^[^ ^26^ ^]^ Recent studies have demonstrated the phenotypic plasticity of the CF through mechanosensitive responses, mediated biochemically by calcium signaling and biophysically by increased *α‐SMA*.^[^ ^27^, ^28^, ^29^ ^]^ Additionally, alterations in stretch magnitude and stiffness of the microenvironment are known to activate CFs into a myofibroblast phenotype.^[^ ^30^, ^31^ ^]^ These studies highlight the numerous mechanisms driving CF activation and underscore the relevance of better understanding the stress responses of the CF, such as cellular senescence, in the context of cardiovascular diseases.^[^ ^32^ ^]^ Cellular senescence and elimination of senescent cells have been examined as a potential therapy in cardiovascular diseases.^[^ ^21^, ^33^, ^34^ ^]^ Senescent cardiomyocytes display contractile dysfunction due to reduced cellular contractions which result in an increased contractile frequency.^[^ ^21^ ^]^ Nested between the cardiomyocytes, the CFs passively experience this contractile dysfunction, suggesting a continuous crosstalk between cardiomyocytes and CFs.^[^ ^35^ ^]^ Moreover, senescent CFs have been observed in cardiac tissue several days after myocardial infarction which led us to hypothesize about the mode of senescent induction in CFs especially after both a mechanically and biochemically‐stressed event.^[^ ^36^, ^37^ ^]^

To study how contractile dysfunction influences the CF phenotype, we use a reductionist approach to mimic the mechanical disruption described in cardiac disease using an in vitro model. Specifically, the objective of this study was to determine if perturbed mechanical stimulation can induce a premature stress‐induced phenotype in primary murine CFs by examining established markers of cellular senescence. We further investigated the senescent response of CFs through oxidative stress and CFs with a lamin A/C mutation, as alterations in structural integrity of the nuclear envelope and increased oxidative stress are associated with several types of cardiovascular disease.^[^ ^38^, ^39^, ^40^ ^]^ Analysis of the mechanoresponsive nature of the CF revealed that a premature senescent phenotype can be initiated from perturbations in the mechanical environment alone and is unique when compared to chemical induction.

## 2 Results

### 2.1 Perturbed Mechanical Stimulation Initiates a Senescent‐Like Phenotype

**Figure 1:**
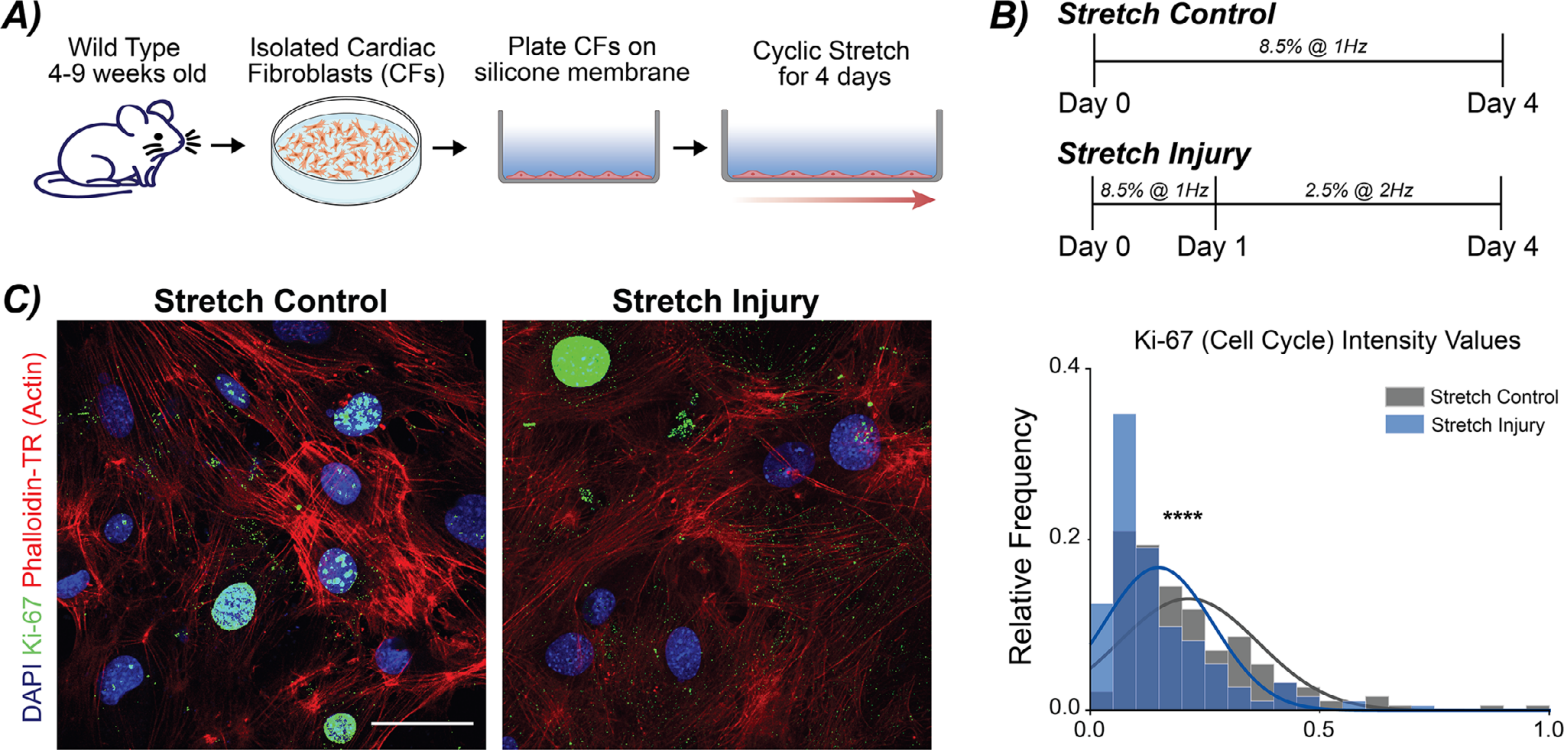
Alterations in mechanical loading disrupts cell cycle and proliferation in primary cardiac fibroblasts. A) Primary murine cardiac fibroblasts (CFs) were isolated from WT mice between 4–9 weeks of age. Cells were expanded on TCP before plating on Geltrex‐coated silicone membranes. CFs were cyclically stretched for 4 days with two separate loading schemes. B) Two loading schemes were selected to mimick the transition in mechanical stretch and frequency changes observed in cardiovascular disease. C) Representative images show Ki‐67 staining of Stretch Control and Stretch Injury cultures. The histogram depicts the relative frequency of Ki‐67 staining between the two loading schemes. A decrease in Ki‐67 positive cells was observed with Stretch Injury, noting a decrease in proliferation. DAPI = blue (405 nm), Ki‐67 = green (488 nm), Actin (Phalloidin‐TR) = red (561 nm). Scale bar = 50 µm. N = 5 animals, n ≥ 25 nuclei/treatment. ****p < 0.0001.

To examine the role that perturbed mechanical stimulation has on cardiac fibroblasts (CFs), we plated primary murine wild type (WT) CFs on flexible membranes and subjected them to two modes of cyclic stretch for 4 days (**Figure** **1**A). The first mode, termed Stretch Control, was 8.5% strain at 1 Hz with daily medium changes. The second mode, termed Stretch Injury, which mimicked altered mechanical loading observed in cardiac disease^[^ ^30^, ^31^ ^]^ was 24 h at 8.5% strain at 1 Hz, and then the strain was decreased to 2.5% strain and frequency increased to 2 Hz (Figure 1B). This decreased strain and increased frequency loading mode was selected to mimic the contractile dysfunction observed in cardiac disease in both humans and mice.^[^ ^22^, ^41^ ^]^ After 4 days (96 h), stretched cells were analyzed with gene expression, immunofluorescence for markers of cell cycle arrest, alterations in the nuclear envelope, and DNA damage. When comparing Stretch Control CFs to Stretch Injury CFs, we observed a decrease in proliferation. Specifically, using Ki‐67 that labels cells in G1, G2, and S phase of the cell cycle, Stretch Injury CFs showed a significant decrease in the Ki‐67 normalized intensity values compared to Stretch Control CFs (Figure 1C), consistent with reduced proliferative capacity and cell cycle arrest, a hallmark of senescence.

**Figure 2:**
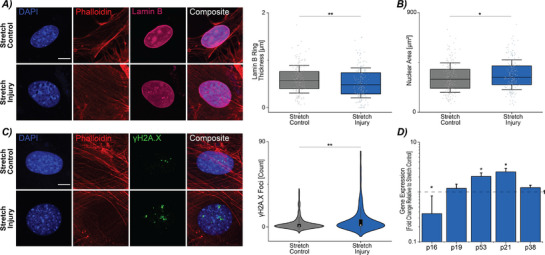
Perturbed loading of cardiac fibroblasts triggers markers indicative of a mechanically‐induced senescent phenotype, including alterations in morphology, DNA damage, and gene expression. A) Cyclically stretched CFs in the control and injury loading schemes were stained for lamin B. Expression of lamin B was assessed by quantifying the ring thickness around the nucleus with Stretch Injury cultures having a decrease compared to Stretch Controls. Scale bar = 10 µm. Error bar = 1 Std. B) Stretch Injury CFs had a decrease in nuclear area compared to Stretch Control. Error bar = 1 Std. C) γH2A.X foci were counted per nucleus between the two loading schemes. Stretch Injury CFs had increased foci compared to stretch controls. Error bar = 1 Std. Scale bar = 10 µm. For A‐C, N = 7 animals, n ≥ 25 nuclei/treatment. D) Gene expression analysis showed a significant increase in p53 and p21 in Stretch Injury CFs. N = 9 animals. Error bar = sem. Linear mixed model, ANOVA, **p < 0.01, *p < 0.05.

Next, we assessed Stretch Injury CFs for other hallmarks of senescence such as lamin B expression, alterations in nuclear morphology, DNA damage, and gene expression for markers of cell cycle arrest. Decreased lamin B is observed in senescent cells both in vivo and in vitro.^[^ ^42^ ^]^ To evaluate lamin B levels through immunofluorescence, we quantified the thickness of the lamin B ring as a surrogate measure of lamin B protein expression. Stretch Injury CFs had a significantly decreased lamin B ring compared to Stretch Control CFs (**Figure** **2**A). Additionally, nuclear area significantly increased in Stretch Injury CFs (Figure 2B); however, changes in nuclear aspect ratio were not observed (Figure S1A, Supporting Information). Surprisingly, Stretch CFs had increased chromatin condensation and H3K9me3 foci, even though other studies have shown chromatin relaxation and decreases in H3K9me3 with senescent phenotypes^[^ ^43^ ^]^ (Figure S1B,C, Supporting Information). Next, we assessed DNA damage in Stretch Injury CFs for γH2A.X foci, a marker of double‐stranded breaks (DSB) commonly observed in stress‐induced premature senescence.^[^ ^5^ ^]^ Using a custom MATLAB code, we quantified the number of γH2A.X foci per nucleus for each stretch mode. By measuring DNA damage through the presence of γH2A.X, we found a significant increase in γH2A.X foci per nucleus in the Stretch Injury CFs compared to the Stretch Control CFs (Figure 2C). The increased γH2A.X in Stretch Injury CFs could provide one signaling mechanism that triggers the initiation of the senescent state.

We further investigated gene expression for markers of cell cycle arrest, p16, p19, p21, and p53, in the stretched cultures via RT‐qPCR. The p53‐p21‐RB axis is known to be involved in DNA damage response signaling and cell cycle arrest.^[^ ^44^, ^45^ ^]^ Stretch Injury CFs had increased expression of p53 and p21 but surprisingly had decreased expression of p16 (Figure 2D). Together, we found mechanical perturbation of primary murine CFs induced a senescent‐like phenotype when assessed through markers of cell cycle, nuclear morphology, and DNA damage.

### 2.2 Chemical and Mechanical Induction of Senescence have Unique Pathways

**Figure 3:**
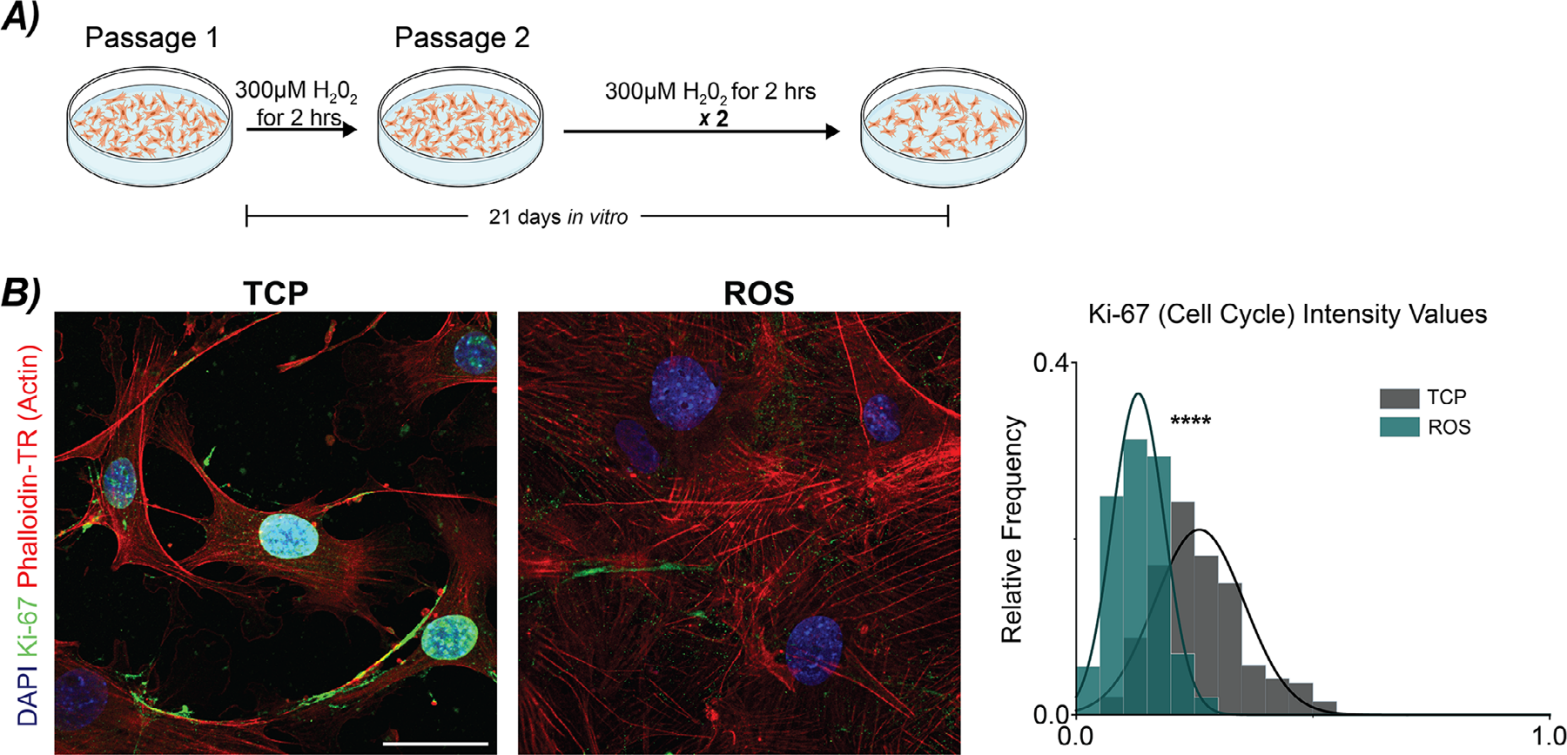
Validating markers of mechanically‐induced senescence through reactive oxygen species (ROS)‐induced senescence. A) Isolated CFs from 4–9 week old WT mice were plated on TCP and treated with 300 µm H_2_O_2_ for 2 h over the course of 21 days in vitro to induce a senescent phenotype. B) Representative images show Ki‐67 staining of CFs in TCP and ROS cultures. Quantification of Ki‐67 positive cells in TCP and ROS cultures indicated a decreased relative frequency of cycling cells in ROS cultures, noting a decrease in proliferation. DAPI = blue (405 nm), Ki‐67 = green (488 nm), Actin (Phalloidin‐TR) = red (561 nm). N = 7–8 animals, n ≥ 25 nuclei/animal/treatment. Scale bar = 50 µm. Linear mixed model, ANOVA. ****p < 0.0001.

**Figure 4:**
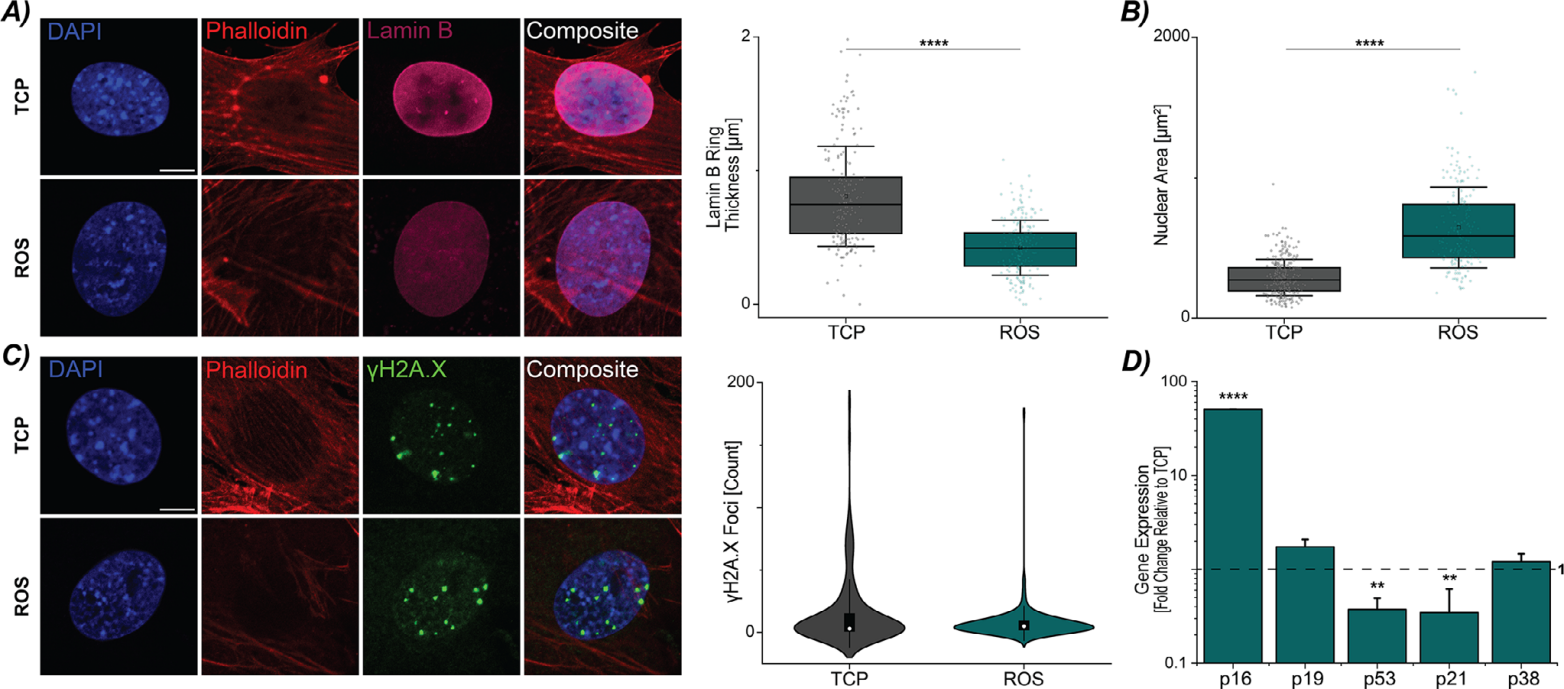
Cardiac fibroblasts under oxidative stress exhibit markers indicative of a chemically‐induced senescent phenotype, including alterations in morphology, DNA damage, and gene expression. A) Representative images of lamin B staining from CFs in TCP and ROS cultures. Cells in ROS cultures showed a decrease in the lamin B ring thickness. Scale bar = 10 µm. Error bar = 1 Std. N = 7–8 animals, n ≥ 25 nuclei/treatment. B) ROS‐induced CFs had an increase in nuclear area. Error bar = 1 Std. N = 8–10 animals, n ≥ 25 nuclei/treatment. C) Representative images from TCP and ROS cultures showing γH2A.X foci. Nuclei from CFs in ROS cultures did not show increased γH2A.X foci compared to TCP CFs. Error bar = 1 Std. Scale bar = 10 µm. N = 6–8 animals, n ≥ 25 nuclei/treatment. D) Gene expression analysis of ROS cultures has increased p16 expression, and decreased p53 and p21 expression. N = 8 animals. Linear mixed model, ANOVA. ****p < 0.0001, **p < 0.01.

Given that a combination of markers is necessary to define a senescent phenotype and several markers are not senescent exclusive, we sought to additionally induce senescence through oxidative stress, reactive oxygen species (ROS)‐induced senescence, which is observed in cardiovascular disease, especially after injury.^[^ ^40^ ^]^ We cultured primary WT CFs over 21 days with hydrogen peroxide treatment to chemically induce senescence (**Figure** **3**A). We then assessed ROS‐induced CFs for the same markers as the mechanically‐induced CFs. We found the ROS‐induced CFs had a significant decrease in Ki‐67 staining compared to TCP controls indicating decreased proliferation. Using the same quantification of lamin B ring thickness, we found a decrease in ring thickness (**Figure** **4**A) in ROS‐induced CFs and a general decrease in lamin B intensity as shown in the representative images. Analyzing morphological changes, we observed a significant increase in nuclear area in ROS‐induced CFs (Figure 4B). Interestingly, while we observed decreases in the aspect ratio and chromatin condensation, this finding is opposite to the phenotype detected with the mechanically‐induced senescent CFs where we observed increased aspect ratio and chromatin condensation (Figure S2B,C, Supporting Information).

We examined the ROS‐induced senescent CFs for increased DNA damage through γH2A.X foci count, and we found no difference between TCP and ROS‐induced CFs (Figure 4C). The lack of a significant difference in γH2A.X foci was supported by gene expression analysis for p53 and p21 in which a decrease in both genes were observed compared to TCP controls (Figure 4D). Similar to mechanically‐induced senescent CFs, we observed no difference in p38 gene expression. However, ROS‐induced CFs showed an increase in p16 expression. Taken together, the mechanically‐induced senescent‐like CFs display similar hallmarks of senescence as ROS‐induced CFs with decreased proliferation (i.e., Ki‐67), decreases in lamin B, and increases in nuclear area. However, we found the modes of induction could be unique and dependent on the mechanical versus biochemical stimulus. While both treatments showed increased expression of cell cycle arrest markers, ROS‐induced CFs exhibited significant upregulation of p16, whereas mechanically‐induced CFs had increases in p53 and p21 expression.

### 2.3 Loss of Nuclear Structural Integrity does not Enhance the Initiation of a Senescent State

**Figure 5:**
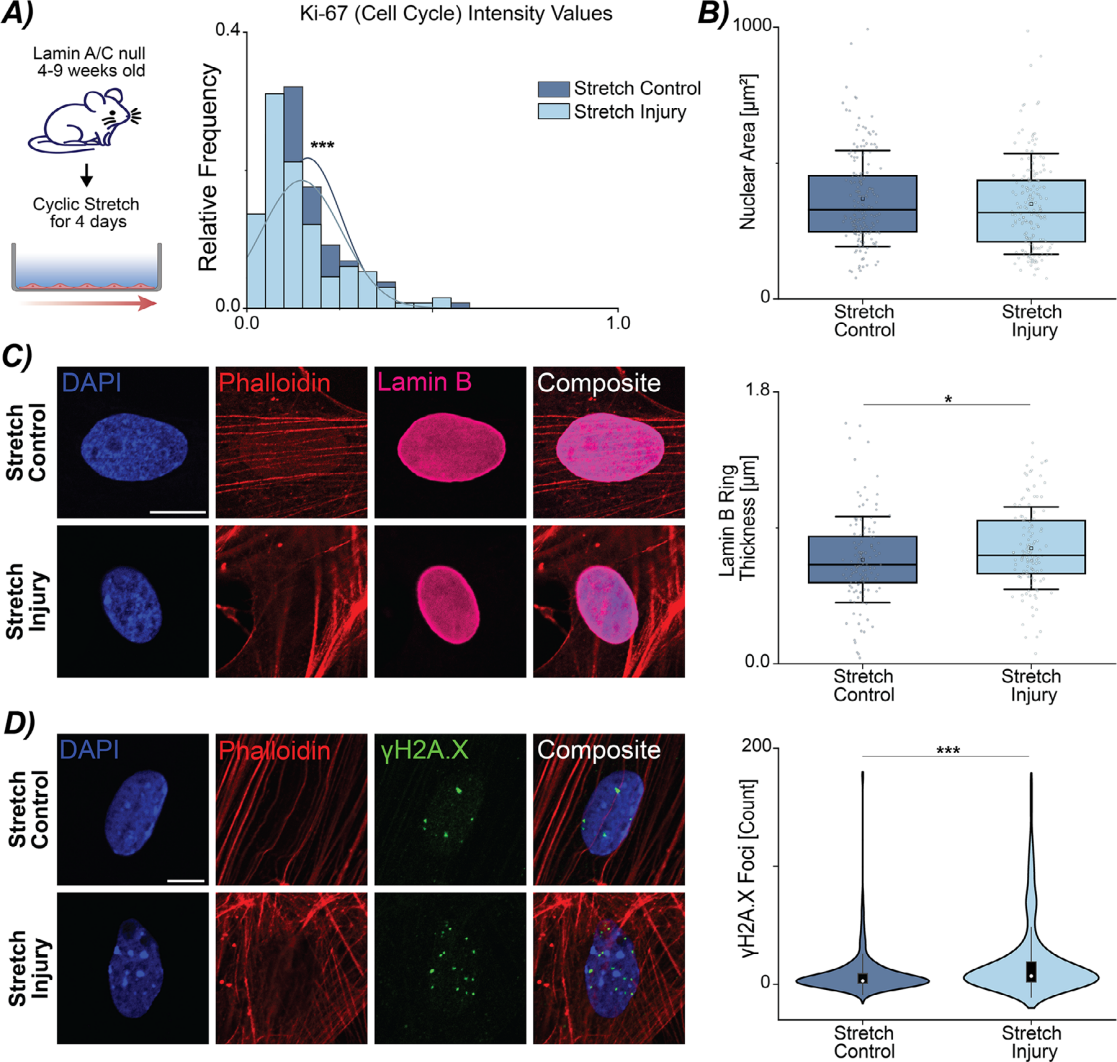
Disruption of nuclear integrity through loss of lamin A/C does not amplify a premature mechanically‐induced senescent phenotype in cardiac fibroblasts. A) Isolated CFs from 4–9 weeks old lamin A/C null mice subjected to Stretch Injury have decreased Ki‐67 positive cells compared to Stretch Control cells. N = 5 animals. B) Mechanical stretch does not alter nuclear area in Stretch Injury CFs compared to Stretch Control CFs. N = 7 animals. C) In lamin A/C null CF nuclei, Stretch Injury loading results in an increase in lamin B thickness compared to Stretch Control. N = 5 animals. D) Stretch Injury CFs have an increase in γH2A.X foci. N = 7 animals. Scale bar = 10 µm. B–D: Error bar = 1 Std. A‐D: n ≥ 25 nuclei/treatment. Linear mixed model, ANOVA. ***p < 0.001, *p < 0.05.

As many alterations and markers found in senescent cells overlap with changes in the nucleus and nuclear envelope, we hypothesized that mechanically‐sensitive proteins in the nuclear envelope might amplify the initiation of the senescent‐like phenotype similar to the profiles characterized in the WT stretch cultures. We used a mouse model where the lamin A/C gene is modified resulting in a loss of the A‐type lamins at the nuclear periphery and integration with the nuclear envelope.^[^ ^46^ ^]^ Lamin A/C maintains the mechanical tension of the nuclear envelope and has been shown to interact with p16 and p53.^[^ ^47^ ^]^ We established primary cultures of CFs from lamin A/C null mice between the ages of 4–9 weeks as mice with the mutation are severely runted and die of cardiac failure around 9 weeks. Next, we evaluated the cultures under the same two loading conditions, Stretch Control and Stretch Injury. We first assessed the cultures for Ki‐67 expression. We observed a significant decrease in the Ki‐67 distribution in Stretch Injury CFs compared to Stretch Control CFs (**Figure** **5**A; Figure S3A, Supporting Information), which was similar to what we observed in Figure 1A. Examining the Stretch Control and Injury lamin A/C null CFs for the defined morphological markers of senescence, we observed no change in nuclear area (Figure 5; Figure S3B, Supporting Information) or aspect ratio (Figure S3E, Supporting Information). However, we found an increase in chromatin condensation (Figure S3D, Supporting Information), similar to the WT mice (Figure S1B, Supporting Information). Interestingly, Stretch Injury lamin A/C null CFs had increased lamin B ring thickness compared to Stretch Controls (Figure 5C, Supporting Information), possibly as a compensatory mechanism in the absence of lamin A/C. While we did not observe a decrease in lamin B ring thickness, which would confirm a senescent‐like phenotype, we noted an increase in nuclear rupture and regions of lamin B dilution in the mechanically‐stimulated (Stretch Control and Injury) lamin A/C null CFs suggesting decreased structural integrity by the loss of lamin A/C along the nuclear envelope. This rupture occurred at the site of lamin B dilution but not at the highest area of curvature within the CF nucleus (Figure S4A, Supporting Information).^[^ ^48^ ^]^ Examining the DNA damage response in the CFs, we observed increase γH2A.X foci in the Stretch Injury lamin A/C null CFs (Figure 5D). However, gene expression analysis did not show significant differences in p53 or p21, which were both upregulated for the WT mechanically‐induced senescent CFs (Figure S3B, Supporting Information).

Recognizing that due to the interaction between lamin A/C and p16^[^ ^47^ ^]^ a senescent phenotype may again be unique, we performed the hydrogen peroxide treatment of the lamin A/C null CFs to generate a ROS‐induced senescent phenotype. Besides changes in lamin B thickness, ROS‐induced lamin A/C null CFs displayed similar hallmarks of senescence as WT CFs (Figure S5, Supporting Information). This again demonstrated the necessity for describing several markers to characterize the senescent phenotype, as lamin B ring thickness did not decrease in the mechanically‐ or chemically‐induced lamin A/C null CFs. Taken together, the compilation of morphological readouts, gene expression, and DNA damage did not indicate the progression of a premature senescent state amplified by altered nuclear structural integrity through the loss of lamin A/C.

### 2.4 Mechanically‐Sensitive Protein, Emerin, Decreases with Stretch Injury

**Figure 6:**
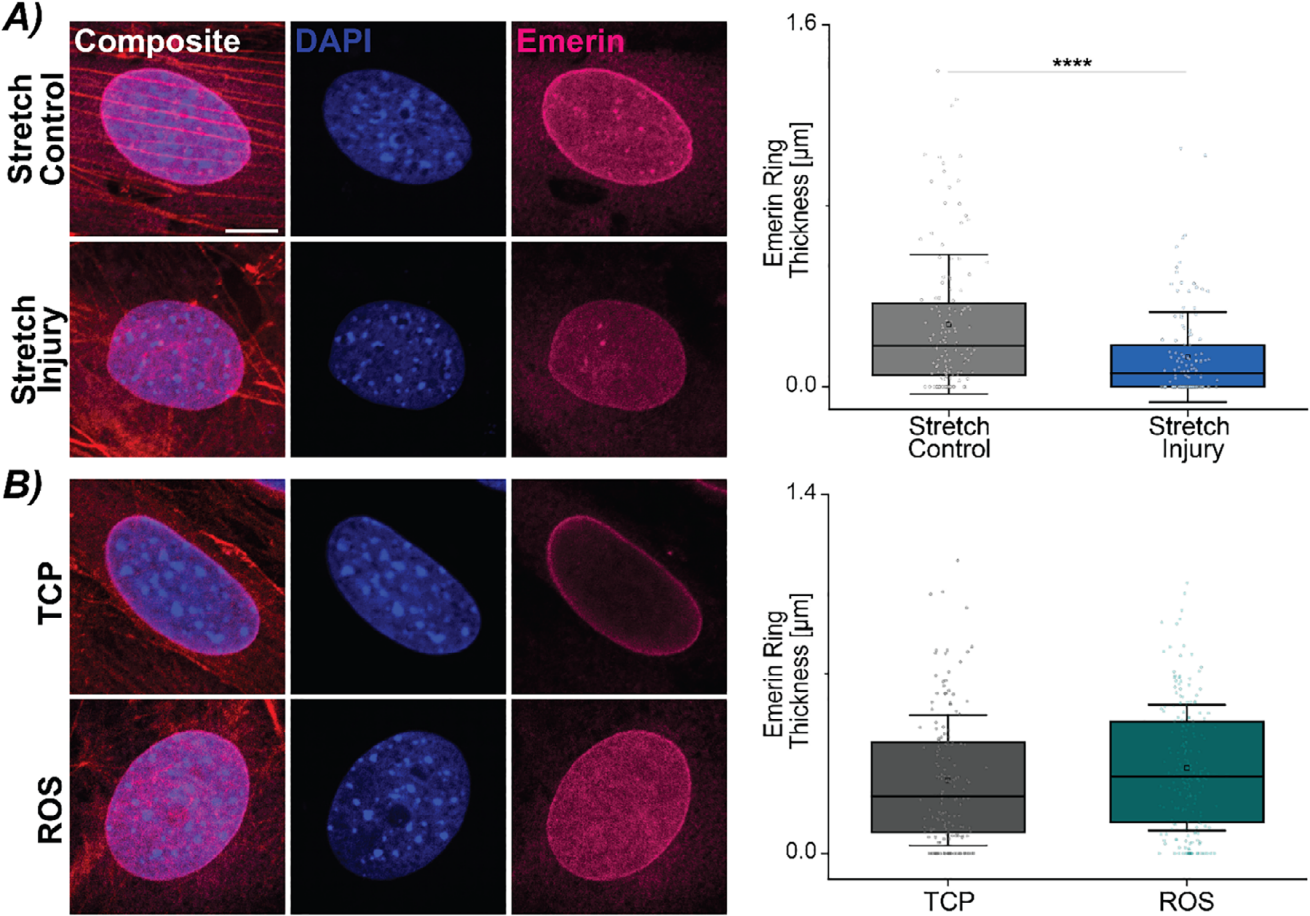
Mechanosensitive nuclear envelope protein, emerin, may be a key mediator in the initiation of a mechanically‐induced senescence phenotype in cardiac fibroblasts. Representative images show emerin staining for nuclei from WT CFs from mechanically‐induced, chemically‐induced, and respective control cultures. Scale bar = 10 µm. A) Quantifying the emerin ring thickness in Stretch Control and Stretch Injury CFs indicated a decreased ring in Stretch Injury CFs. N = 5 animals. n ≥ 25 nuclei/treatment. B) Emerin ring thickness between TCP and ROS‐induced CFs showed no significant alterations with the induction of a senescent phenotype. N = 6–8 animals, n ≥ 25 nuclei/treatment. Error bar = 1 Std. Linear mixed model, ANOVA. ****p < 0.0001.

Since we did not observe an exaggerated phenotype with the lamin A/C null CFs, we looked at other mechanically‐sensitive proteins in the nuclear envelope. Emerin is a nuclear envelope protein that tightly interacts with lamin A/C in regulating nuclear mechanics.^[^ ^49^, ^50^ ^]^ Studies have found that increased strain can cause emerin to locate to the outer nuclear membrane.^[^ ^51^ ^]^ Using the same stretching protocol to initiate a mechanically‐induced senescent phenotype (Figure 1B), we next examined emerin using immunofluorescence. We found Stretch Injury CFs had a significantly decreased emerin ring thickness compared to Stretch Control CFs (**Figure** **6**A). Curious if this finding was maintained by all senescent CFs, we quantified the emerin ring thickness in ROS‐induced and TCP CFs, and did not find alterations with chemically‐induced senescent CFs (Figure 6B). In all lamin A/C null treatments, mechanically‐ and chemically‐stimulated, we measured decreased emerin ring thickness but no significant differences were found between the treatments (Figure S6A,B, Supporting Information). This is not unexpected as emerin localization in lamin A/C null CFs has been shown to be largely localized to cytoplasm.^[^ ^46^ ^]^ Additionally, with the decreased emerin ring thickness, we also qualitatively observed irregular actin structural patterns immediately surrounding the nuclear envelope. Together, these results point to emerin localization as a distinguishing feature of mechanically‐induced senescence, highlighting its role as a mechanosensitive regulator of nuclear structural integrity and its interplay with the induction of a senescent phenotype.

## 3 Discussion

In this study, we examined how perturbed mechanical stimulus can aid in the initiation of cellular senescence in a simplified in vitro model of cardiac disease. Using previously established markers of cellular senescence, we found that perturbed mechanics can alter proliferation, lamin B expression, induce a DNA damage response, and increases in p21 and p53 expression to define a mechanically‐induced senescent profile in CFs. Furthermore, we found emerin, a mechanically‐sensitive protein in the nuclear envelope may be involved at the initiation stage.

### 3.1 Mechanical Stimulation and Cellular Senescence

With continued growth in the fields of aging and cellular senescence, the factors that induce the transition toward a senescence phenotype are becoming more well‐defined. This adds to the complexity in defining this cellular state as there is still no single marker of cellular senescence.^[^ ^10^ ^]^ Instead, numerous markers that encompass both morphological and biochemical responses are necessary to determine the phenotype.^[^ ^6^ ^]^ Additionally, the markers associated with the induction of cellular senescence can vary within the same cell type, depending on the type of stimulation and the activated pathway. This heterogeneity occurs even at a single cell level.^[^ ^6^, ^45^, ^52^ ^]^ Among the various mechanisms being explored, mechanical stimulation is increasingly being recognized as a key factor in triggering cellular senescence.

In diseases such as intervertebral disc degeneration and osteoarthritis, increases in dynamic cyclic loading using nucleus pulposus cells or chondrocytes have been shown to induce premature senescence through DNA damage response signaling and the p53/p21 axis, but not p16,^[^ ^11^, ^53^ ^]^ similar to our results. Interestingly, in nucleus pulposus cells, high‐magnitude compression also triggered cellular senescence further supporting that abnormal mechanical loading can drive this phenotype.^[^ ^54^ ^]^ Additionally, studies have examined how the stiffening of the local microenvironment can trigger alterations.^[^ ^12^ ^]^ Taken together, these studies demonstrate that perturbed mechanical stimulation can be one of the initial factors in the transition. However, in these studies, a significant increase in strain magnitude (at least 4‐fold higher) or overloading of the cells is frequently used to stimulate a senescent state.^[^ ^11^, ^12^ ^]^ Interestingly, and in contrast, our results showed a decrease in (i.e., hypophysiologic) strain magnitude and increased cyclic frequency as a key driver for the senescent transition indicating that maybe any alteration in cellular mechanics outside of “normal” physiological loading could initiate senescence.

We were surprised to observe senescence was initiated through the activation of distinct pathways, resulting in differences in the cellular senescent phenotype within the same cell type. Through single‐cell phenotyping, we distinguish pathway‐specific patterns of senescence that can be masked in bulk measurements.^[^ ^6^ ^]^ Comparing WT Stretch Injury CFs to those activated through oxidative stress (ROS‐induced) identified two unique senescent profiles indicating differing pathways of activation. ROS‐induced CFs had increased expression in p16, but we observed no differences in DNA damage through γH2A.X foci compared to CFs on TCP. Stretch Injury CFs had increased p21 and p53 gene expression, and an increased DNA damage response compared to Stretch Control CFs. Both modes of induction led to reduced lamin B ring thickness and Ki‐67 expression. Similarly, lamin A/C null mechanically‐stimulated CFs and ROS‐induced senescent CFs displayed distinct phenotypes, distinguished by variations in gene expression and lamin B expression through quantitative assessment of ring thickness. This difference could be explained by the mechanical stimulation the Stretch Injury CFs experience. The increase in lamin B could be a compensatory mechanism for the lack of lamin A/C, which is known to regulate nuclear tension.^[^ ^55^, ^56^ ^]^

### 3.2 Nuclear Integrity and Mutations in Lamin A/C

Lamin A/C is a nuclear envelope protein that supports the nucleus both structurally in maintaining shape and size, but additionally interacts with genes necessary for cell cycle and DNA damage repair.^[^ ^47^, ^57^ ^]^ There is an array of diseases associated with the mutations in the *Lmna* gene, termed laminopathies. Depending on the mutation or deficiency, phenotypic effects range from diseases which cause accelerated aging as seen in Hutchinson‐Gilford Progeria Syndrome, to those which result in severe retardation of skeletal muscle and cardiac disease as observed in Emery‐Dreifuss muscular dystrophy (EMDM).^[^ ^39^ ^]^ The large‐scale effects resulting from disruption in the nuclear envelope show the importance of the nuclear envelope and its integrity for healthy cell and tissue maintenance.^[^ ^58^ ^]^ A complete deficiency in lamin A/C in the nuclear periphery has been shown to lead to altered chromatin organization and increased nuclear fragility, nuclear rupture, blebbing, and DNA damage.^[^ ^55^, ^59^, ^60^ ^]^ As lamin A/C helps to regulate nuclear tension, it has also been shown to be mechanically sensitive to both gradual changes in the microenvironment and applied forces.^[^ ^8^, ^55^ ^]^ In this study, we found that alterations in the nuclear envelope, specifically with the knockout of lamin A/C, led to increased DNA damage within the nucleus and severe nuclear ruptures within the stretched cultures. In the mechanically stretched lamin A/C null CF cultures, nuclear blebbing was not observed. However, the lamin A/C null CFs plated on TCP, both TCP only and ROS‐induced, showed nuclear blebbing confirming the morphological perturbations as described in literature.^[^ ^56^ ^]^ Interestingly, an increase in lamin B thickness was observed in lamin A/C null CFs including the CFs with nuclear rupture. In the lamin A/C null CFs, lamin B may additionally act as a compensatory mechanism in nuclear maintenance of both shape and size.

Lamin A/C has been shown to interact with p16 and disruption of this interaction alters the cell cycle.^[^ ^47^ ^]^ In mechanically stretched cultures, lamin A/C null CFs did not show increased p53/p21 or p16 even though a decrease in Ki‐67 was observed. It remains uncertain whether the reduced proliferation reflects cell cycle arrest at the gene expression level or instead resulted from increased cell death via apoptotic pathways. Future experiments looking at apoptosis in the lamin A/C null CFs and increased mechanical stimulation could be performed to clarify the difference observed between gene expression and Ki‐67 staining.

Comparing mechanical stimulation in WT versus lamin A/C null CFs, we did not detect a senescent phenotype in the lamin A/C null CFs using the same senescent markers. Both phenotypes exhibited significant increases in chromatin condensation and γH2A.X foci, along with decreased proliferation as measured by Ki‐67. However, nuclear area and lamin B ring thickness showed contrasting changes. Since both these measures reflect nuclear architecture, it is not unexpected that the absence of lamin A/C resulted in opposing observations. In contrast, chemical induction of senescence yielded more consistent effects between the WT and lamin A/C null CFs. With the exception of the lamin B ring thickness, similarities were observed in proliferation, nuclear area, and gene expression. Future experiments examining SASP factors or lysosomal activity could be used to help identify additional shared markers that could better identify a senescent phenotype in lamin A/C‐deficient cells.

### 3.3 Nuclear Integrity and Emerin

Within the nuclear envelope, other proteins have been identified as mechanically sensitive, for example, the linker of nucleoskeleton and cytoskeleton (LINC) complex and emerin.^[^ ^8^, ^50^, ^61^ ^]^ Disruptions in emerin also have large‐scale effects in development and is another known cause of muscular dystrophy. Primarily located on the inner nuclear membrane, emerin transfers information between the cytoskeleton (i.e., microtubules and F‐actin) and the nucleus. Moreover, emerin interacts with not only with the nuclear envelope proteins but also interacts with lamin A/C and lamin B, and binds to actin to maintain chromatin architecture, nuclear shape, and aspect ratio.^[^ ^49^, ^50^, ^62^ ^]^ Disruption or complete knockdown of emerin shows changes in F‐actin patterning, impaired actin binding, and nuclear alignment.^[^ ^49^, ^62^ ^]^ Alterations in strain and actin polymerization have been noted following stretch injury,^[^ ^63^ ^]^ and separately have shown increases of emerin in the outer nuclear membrane.^[^ ^51^ ^]^ Our results agree with these findings showing alterations in the localization of emerin within the nuclear envelope and cytoplasm of the CF. The lack of an inner emerin ring in the lamin A/C null cultures, both mechanical and chemical, and Stretch Injury CFs indicates that emerin acts as a key element in maintenance of strain transfer with active mechanical forces. Future studies could examine emerin's interaction with actin and localization within senescent CFs as potential therapeutic target. Indeed, regulation of the nuclear envelope has already been implicated in mechanically‐stressed induced senescence in skeletal muscle MSCs, which indicates a significant connection between induction of cellular senescence and nuclear integrity.^[^ ^64^ ^]^

While this study focused on mechanical stress as a primary initiating factor of senescence, p16/p21/p53 axes integrate a wide array of upstream signals. These include DNA damage, oncogene activation, mitochondrial dysfunction, inflammatory cytokines, and epigenetic remodeling, in addition to oxidative and mechanical stress.^[^ ^3^ ^]^ We highlight that our findings add to this broader regulatory network by establishing altered mechanical stimulation as a distinct input to these senescence pathways.

From our simplified in vitro model of the mechanical perturbations observed in cardiac disease, we found altered mechanical stimulation in CFs leads to a mechanically‐induced premature senescent‐like phenotype. Use of the lamin A/C null model was intended to probe the fundamental mechanobiological regulation of senescence and the results highlight nuclear envelope proteins as key modulators. The study demonstrates how hypophysiologic changes in mechanical stimulation, and more specifically, a decrease in strain and increased frequency can initiate cellular senescence. Additionally, we identify a set of markers to characterize a mechanically‐induced phenotype advancing the effort to more precisely define cellular senescence.

## 4 Experimental Section

### Cardiac Fibroblast Cultures

Primary murine cardiac fibroblast (CF) cultures were derived from B6.129S1(Cg)‐*Lmna^1Stw^*/BkknJ wild type (WT) and null mice (Jackson Labs, Stock No.: 0 09125).^[^ ^46^ ^]^ The study included mice between the ages of 4–9 weeks with no exclusion of sex. Specific pathogen‐free, temperature‐controlled housing with 12‐h light cycles maintained the mice, which received food and water ad libitum. All animal procedures followed University of Colorado Boulder Institutional Animal Care and Use Committee approval (2628‐1). Cardiac tissue was excised from mice and placed immediately in cold DPBS (Hyclone, SH30028.03). A 25‐gauge needle inserted in the apex of the tissue allowed flushing of hearts with 30 mL of cold DPBS. The tissue was then minced with a scalpel and washed three times in ice‐cold HBSS (Gibco, 4500‐462) in a sterile environment. Digestion medium, containing 0.2% w/v collagenase P (Roche, 11 213 873 001) in DMEM supplemented with 3% v/v FBS (Gibco, 261‐40‐079) and 10 µg ml^−1^ DNase1 (Sigma, D4263‐5VL), was added to the minced tissue. The tissue underwent digestion for 25 min at 37 °C at 900 rpm. Following this period, the tissue was triturated ten times with a P1000 tip to mechanically disrupt the tissue. The digested mixture was incubated for an additional 10 min at 37 °C at 900 rpm. After a second mechanical disruption, the tissue was filtered through a 40 µm cell strainer and rinsed with supplemented medium, D10. The cardiac fibroblast medium, D10, consisted of DMEM/F12 (Gibco, 1330‐032) supplemented with 10% v/v FBS (Gibco, 261‐40‐079), 1× Penicillin/Streptomycin (Gibco, 15140‐122), and 1 µM ascorbate‐2‐phosphate (Sigma, 49752‐10G). Addition of 1 mM EDTA (Sigma, 03690‐100) to D10 quenched the digestion reaction. The resulting cell/tissue pellet was lysed with 1× RBC Lysis Buffer (ebioscience, 00‐4300‐54) and the reaction quenched with D10. The cells were plated in 100 mm dishes for 2.5 h in a 37 °C incubator with 5% CO_2_. After 2.5 h, petri dishes were washed three times in warmed PBS and added D10 back to the dishes. Based on previously established methods of isolation and characterization, the remaining cells were deemed adherent to the petri dish cardiac fibroblasts.^[^ ^65^ ^]^

Passage 1 CFs were divided up into 4 uses: RNA, mechanical stimulation, immunofluorescence (e.g., TCP, ROS, mechanical), and continued culture. For tissue culture plastic (TCP) controls for RNA and immunofluorescence (IF), cells were plated at a density of 3 × 10^3^ cm^−2^ in 8 well ibidi (IF) dishes and 6‐well plates (RNA). For mechanical stimulation experiments, both passage 1 and passage 2 cells were used. All CFs used in mechanical stimulation experiments were plated at 6 ×10^3^ cells cm^−2^ on a MCFX 16‐well flexible membrane. Prior to plating, the membranes were coated with Geltrex (Gibco, A1569601) for 16 h at 37 °C. For continued culture CFs, passage 1 CFs were plated at 2.5 × 10^3^ cm^−2^ in 100 mm petri dishes and cultured for 5 days with medium changes every 2 days. On day 5, cells were detached using TrypLE, quenched with D10, and pelleted at 1300 rpm. CF pellets were resuspended in D10 and filtered through 70 µm cell pellet prior to count with Trypan Blue. The CFs were divided into groups for experimental use IF, RNA, and mechanical stimulation. The same coating protocol of Geltrex and plating density was used for P2 CFs subjected to mechanical stimulation. After 4 days of mechanical stimulation, cells were used for RNA and IF. For IF experiments, cells on MCFX plates were fixed in 4% PFA in DPBS for 20 min at room temperature. After 20 min, fixed cells were washed 3 times in DPBS and stored at 4 °C in DPBS until use.

For TCP controls, cells were plated at 3 × 10^3^ cm^−2^ in a 6 well plate. All TCP controls for imaging and IF were collected 3 days after plating. TCP cells used in IF experiments were fixed in 4% PFA in DPBS at room temperature for 10 min. After 10 min, fixed cells were washed in DPBS and stored in at 4 °C in DPBS. For all experiments, RNA was collected by lysing the CFs in Qiazol (Qiagen, 79 306) and stored at −80 °C until extraction.

### Mechanical Stimulation Experiments

Passage 1 and Passage 2 CFs were used for all mechanical stimulation experiments. After plating CFs in the flexible 16 well‐membranes, cells were allowed to adhere for 24 h at 37 °C in 5% CO_2_ before stretching.

### Mechanical Stimulation

Two loading modes were designed: 1) Stretch Control and 2) Stretch Injury. Using a MechanoCulture FX (CellScale, MCFX) device, cells were subjected to uniaxial stretch at different strain magnitudes and rates. The Stretch Control loading scheme was continuously stretched for 4 days at 8.5% strain at a frequency of 1 Hz within an incubator maintained at 37 °C in 5% CO_2_. The Stretch Injury loading scheme was 4 days total with 24 h of mechanical stimulation at 8.5% strain at 1 Hz and 72 h at 2.5% strain at 2 Hz within an incubator maintained at 37 °C in 5% CO_2_ (Figure 1A). Full medium changes were performed every 24 h to decrease any confounding biochemical effects.

### ROS Treatment

Passage 1 cells were plated at 4 × 10^3^ cm^−2^ in a 6 well TCP dish. After 24 h, cells were treated with 300 µM H_2_O_2_ (J.T. Baker, 2186‐01) for 2 h at 37 °C.^[^ ^66^ ^]^ After 2 h, wells were washed once with D10 and cultured in D10 for 48 h. Minimal cells death was observed after the first treatment. After 48 h, the CFs were passed, and all remaining treatments were performed without further passaging. Cells were divided up between RNA and IF experiments after the first treatment. For RNA collection, CFs were plated at 4 × 10^3^ cm^−2^ in a 6 well TCP plate. For IF, CFs were plated at 4 × 10^3^ cm^−2^ in 3.5cm^2^ imaging dishes (ibidi, 50‐305‐806). WT ROS‐induced CFs received a total of three treatments of 300 µM H_2_O_2_ for 2 h at 37 °C. Lamin A/C null ROS‐induced CFs received a total of two treatments of 300 µm H_2_O_2_ for 2 h at 37 °C due to increased cell death observed in the cultures. Culture medium was changed every 3–4 days. RNA and IF dishes were collected 8 days after the final H_2_O_2_. During the 8‐day incubation period, increased cellular and nuclear areas were observed, indicating potential initiation of cellular senescence. These CFs were referred to as ROS‐induced CFs in the results section.

### RNA Isolation and RT‐qPCR

RNA was isolated using Direct‐zol RNA Miniprep (Zymo, 76020–642), nanodropped for concentration and quality, and reverse transcribed into cDNA using iScript Reverse Transcription Supermix (BioRad,1 708 841). Primers were designed using NCBI primer BLAST with all primers designed to span an exon‐exon junction (Table S1, Supporting Informatio). Real‐time quantitative PCR was performed with SsoAdvanced Universal SYBR Green Supermix (BioRad, 1 725 271) in a CFX96 Touch thermocycler (BioRad) using 10 ng of cDNA/reaction. All data was normalized to *Gapdh* prior to fold change. The relative change in gene expression was quantified using the ΔΔCt method.

### Immunofluorescence and Imaging

For immunofluorescence, fixed CFs were permeabilized in 1% Triton‐X in PBS and blocked with 1% v/v normal goat serum with 1% w/v BSA in PBT (0.125% Tween 20 in PBS) prior to staining. CFs were stained with primary antibodies in antibody buffer (1% w/v BSA in PBT) overnight at 4 °C rocking and washed 3 times in PBT to remove any unbound antibody. CFs were incubated in secondary antibodies for 45 min at room temperature. Following two washes in PBT, CFs were stained for DAPI (1:1000; Invitrogen, D1306) and Phalloidin Texas‐Red (1:300, Life Technologies, T7471) for 30 min. ROS CFs and TCP CFs were stored at 4 °C in DPBS until imaged. Due to the thickness of the flexible membrane of the MCFX device, wells were punched out of the flexible plate, flipped, and mounted in DAKO Mounting Medium (Agilent, S302380‐2) for improved resolution for imaging. Primary Antibodies: Mouse anti‐Mouse γH2A.X (1:400, CST, 80312S), Rabbit anti‐Mouse Lamin B1 (1:500, abcam, ab16048), Rat anti‐Mouse Ki‐67 (1:300, ebiosciences, 14‐5698‐82), Rabbit anti‐Mouse Emerin (1:250, CST, 30853S). Secondary antibodies: Goat anti‐Mouse IgG Alexa Fluor 488 (1:500, ThermoScientific, A28175), Goat anti‐Rabbit IgG Alexa Fluor 633 (1:500, ThermoScientific, A21070), Goat anti‐Rat IgG Alexa Fluor 488 (1:100, BioLegend, 405 418).

For imaging acquisition, all fixed and mounted wells were imaged on a Nikon A1R Confocal using 512 × 512 pixel field of view with a 60X oil immersion objective. Greater than 25 nuclei per treatment and genotype were taken. For imaging analysis, a custom MATLAB code analyzed the collected images of the cell nucleus. The process involved cropping, segmenting, and histogram normalizing images for biophysical features. Calculations of lamin B and emerin ring thickness relied on detecting an outer ring, summing the number of pixels around the perimeter of the nucleus based on fluorescence intensity, and then normalizing to the nuclear area. The normalized value was converted to microns using voxel dimensions for an average ring thickness per nucleus. MATLAB's built‐in functions determined other biophysical features such as nuclear area and aspect ratio. Detection of γH2A.X foci employed an intensity threshold method, counting peaks above the set threshold. Proliferation analysis with Ki‐67 involved histogram normalized intensity values examined against relative frequency.

### Statistical Analysis

The difference between treatments (TCP and ROS or Stretch Control and Stretch Injury) were analyzed for lamin B ring thickness, emerin ring thickness, Ki‐67, gene expression analysis, and γH2A.X foci count using linear mixed effects models (nlme package, Version 3.1‐140) in R (RStudio, Version 1.2.1335; R, Version 3.6.1, Boston, MA). Animal was considered a random effect in each model. Type II Sum of Squares ANOVA tested differences between treatments. For γH2A.X foci data, the model was analyzed with and without the outliers to confirm statistical significance and p‐values. Post‐hoc testing utilized the emmeans package with Tukey's HSD corrections for multiple comparisons (Version 1.4.3.01). ANOVA normality assumptions underwent validation through testing the residuals of the model with the Shapiro‐Wilk test and visual examination of qq‐plots. If necessary, data was transformed to meet the normality assumptions of ANOVA. Animal and nuclei number per animal for each treatment analysis are documented in the figure captions with the corrected p‐values for each treatment comparison.

## Conflict of Interest

The authors declare no conflict of interest.

## Author Contributions

S.E.S. and C.P.N. conceptualized the manuscript. S.E.S., A.K.S., E.Y.M., and S.G. performed methodology. S.E.S., A.K.S., and K.G. performed investigation. S.E.S., E.Y.M., and S.G. performed visualization. S.E.S., C.P.N. performed supervision. S.E.S. and C.P.N. wrote the original draft. S.E.S., A.K.S., K.G., E.Y.M., S.G., and C.P.N. wrote, reviewed, and edited the manuscript.

## Supporting information



Supporting Information

## Data Availability

The data that support the findings of this study are available from the corresponding author upon reasonable request.
