# Supplementary material for: Mechanical Stress Triggers Premature Senescence in Cardiac Fibroblasts
Source: Adv Sci (Weinh). 2025 Sep 26;12(47):e13314. doi: 10.1002/advs.202513314 (PMC12713081; doi:10.1002/advs.202513314)
Supplement: Supplementary file 1 — Supporting Information [file ADVS-12-e13314-s001.docx]

**Supplemental Materials**

**
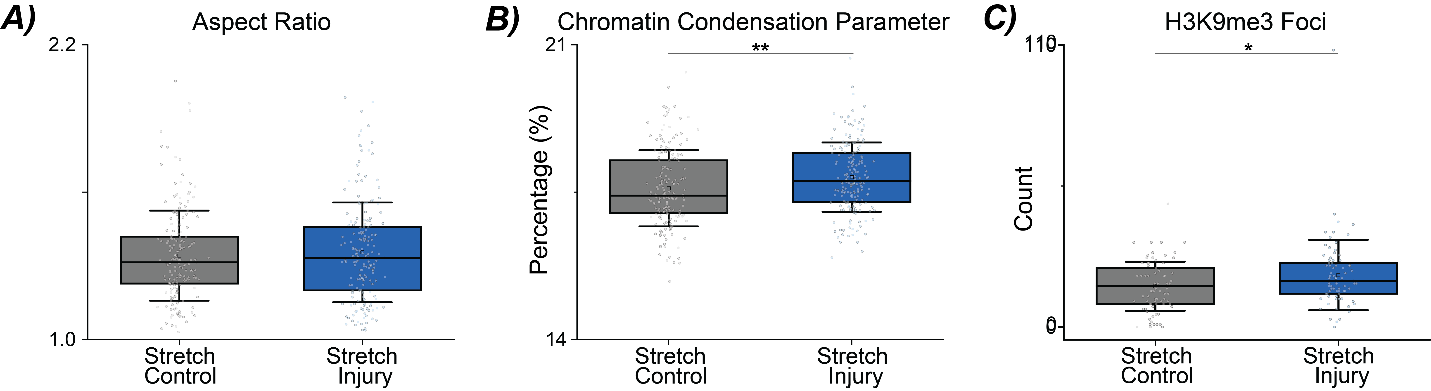
Figure S1. Analysis of the morphological features for mechanically-induced senescent cardiac fibroblasts.** (A) A difference in aspect ratio was not observed when comparing Stretch Control and Stretch Injured CFs. N = 8-10 animals, n ≥25 nuclei/treatment. (B) Alterations in DNA condensation were observed and quantified using Sobel edge detection of DAPI staining in MATLAB. Stretch injured CFs had significantly increased chromatin condensation compared to stretch controls. N = 8-10 animals, n ≥ 25 nuclei/treatment. (C) With increased chromatin condensation, CFs were stained for H3K9me3 in both Stretch Control and Stretch Injury treatment groups. Stretch Injury CFs had a slight increase in H3K9me3 foci compared to Stretch Controls. N = 3 animals, n ≥ 25 nuclei/treatment. Error bar = 1 Std. Linear mixed model, ANOVA. **p<0.01, *p<0.05.

**
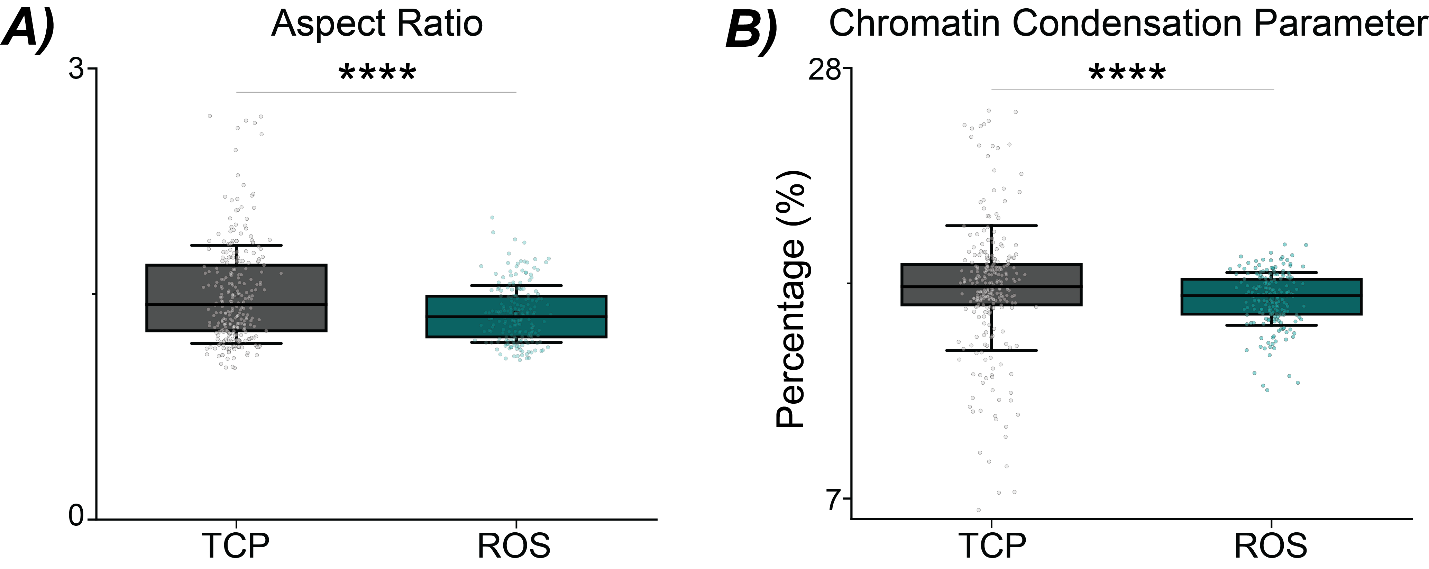
Figure S2. ROS-induced senescent CFs show alterations in nuclear shape and chromatin condensation.** (A) ROS-induced senescent CFs show a significant difference in aspect ratio compared to CFs plated on TCP. (B) Compared to the control TCP CFs, ROS-induced senescent CFs have decreased chromatin condensation. N = 8-10 animals, n ≥ 25 nuclei/treatment. Error bars = 1 std, Linear mixed model, ANOVA. ****p<0.0001.

**
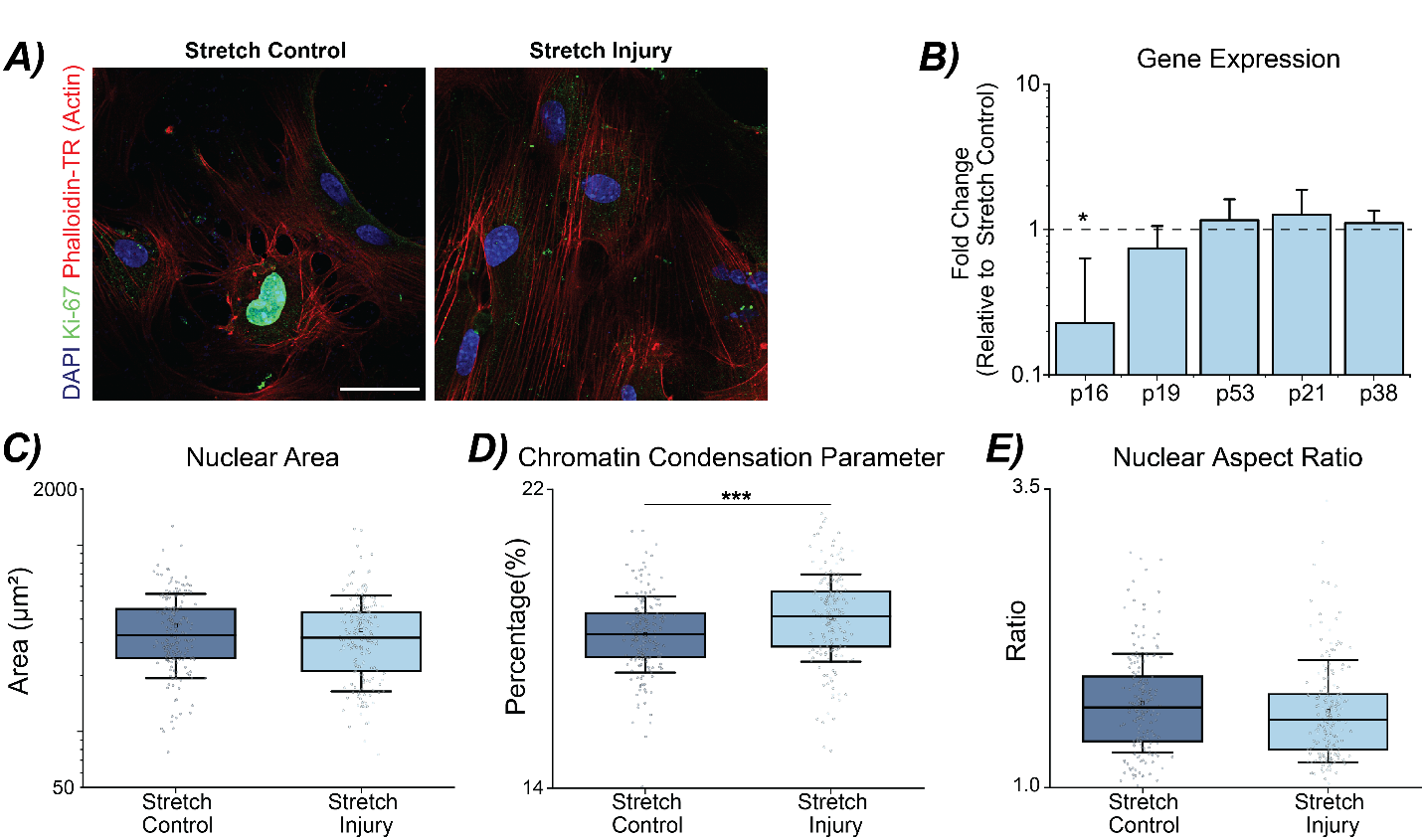
Figure S3. Morphological features of lamin A/C null CFs subjected to Stretch Control and Stretch Injury regimes.** (A) Representative area images of lamin A/C null CFs in Stretch Control and Stretch Injury cultures stained with DAPI (405nm), Ki-67 (488nm), and Phalloidin Texas-Red (Actin, 561nm). N=5 animals, Scale bar = 50µm. (B) Fold change between Stretch Injury lamin A/C null CFs to Stretch Control lamin A/C null CFs showed a significant downregulation of p16. However, no differences were observed with other senescent associated genes. N = 6 animals. Error bar = SEM. *p<0.05. (C). No significant difference was found in nuclear area between Stretch Control and Stretch Injury lamin A/C null CFs. Plot shows log axis of nuclear area (same data as Figure 5B). N = 7 animals, n ≥ 25 nuclei/treatment. Error bar = 1 Std. (D) A significant increase in chromatin condensation parameter was observed in Stretch Injury lamin A/C null CFs. N = 7 animals, n ≥ 25 nuclei/treatment. Error bar = 1 Std. ***p< 0.001. (E) Nuclear aspect ratio does not change with stretch regime for lamin A/C null CFs. N = 7 animals, n ≥ 25 nuclei/treatment. Error bar = 1 Std. Linear mixed model, ANOVA.

**
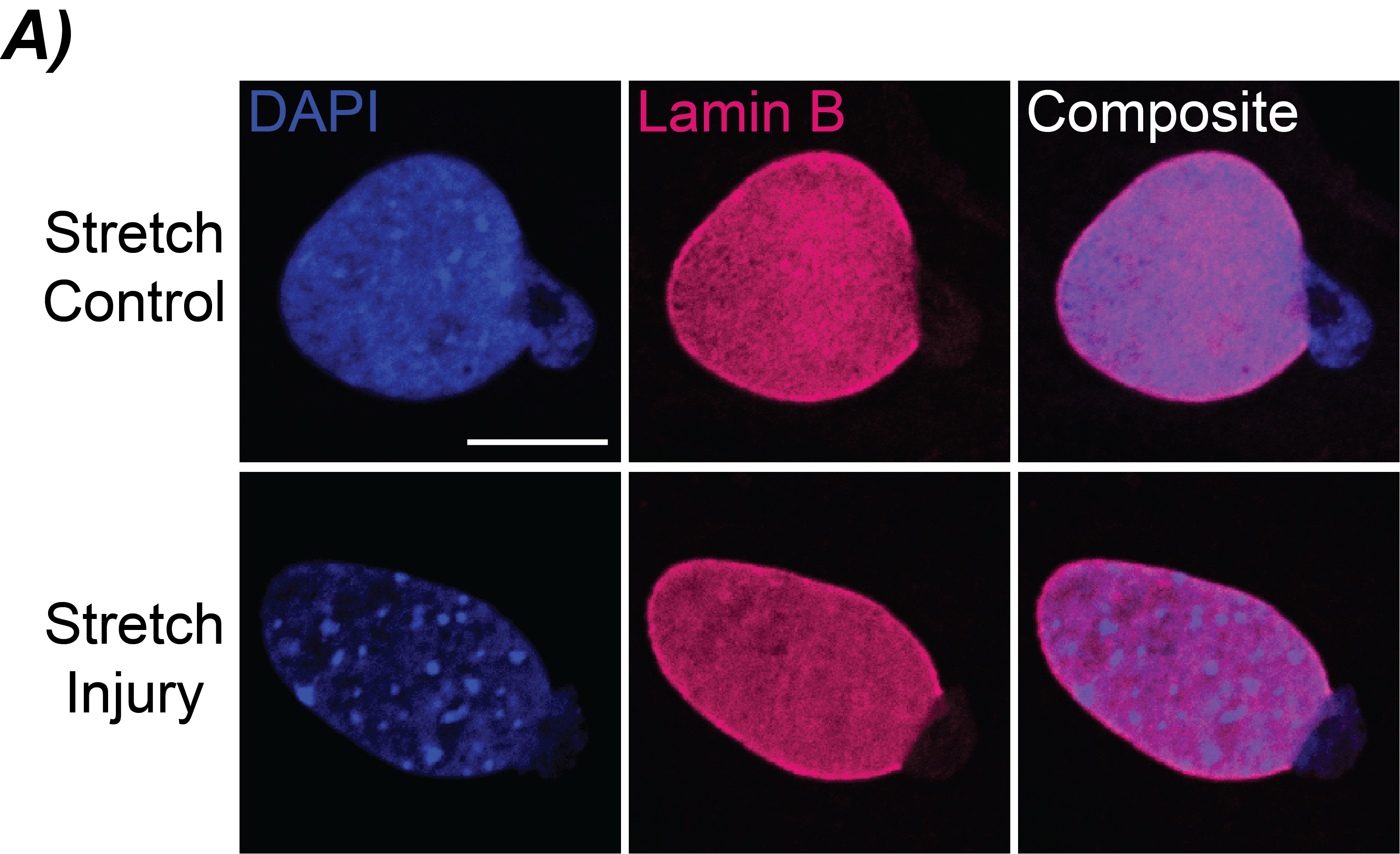
Figure S4. Nuclear rupture observed in mechanical stretched lamin A/C null CFs.** (A) Representative images of Stretch Control and Stretch Injury lamin A/C null CFs. DAPI-stained nuclei show nuclear content expelling from the nucleus which corresponds to the same region of lamin B dilution along the nuclear envelope. Scale bar = 10µm.

**
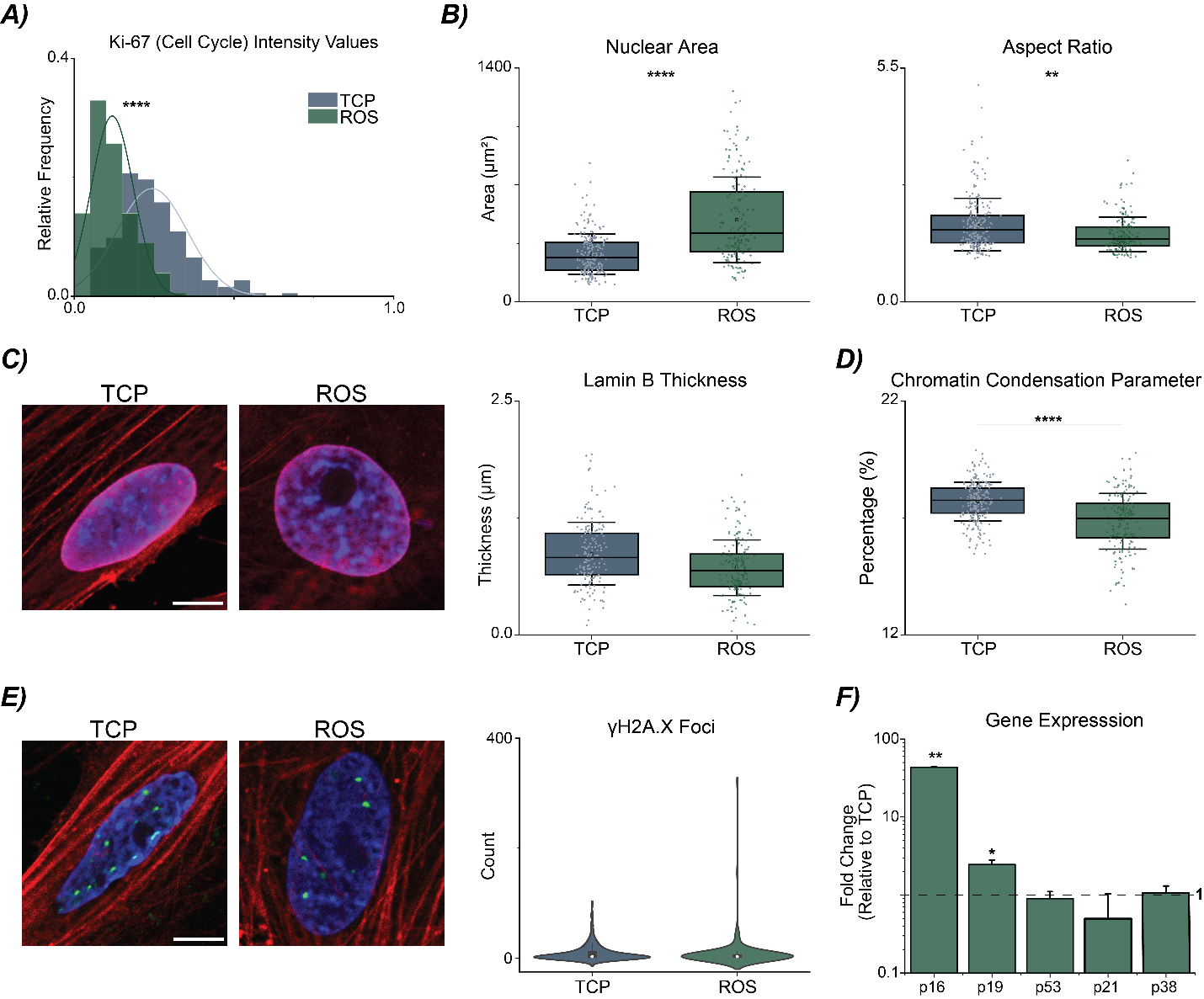
Figure S5. ROS-treated lamin A/C null CFs display several markers of senescence except changes in lamin B thickness and γH2A.X foci counts**. (A) ROS-induced lamin A/C null CFs have a significant decrease in Ki-67 positive cells compared to TCP CFs. N = 7 animals/group, n ≥ 25 nuclei/treatment. (B) Significant alterations in nuclear area and aspect ratio observed between TCP and ROS-induced lamin A/C null CFs have increased nuclear area and a decrease in aspect ratio. N = 7-9 animals/group, n ≥ 25 nuclei/treatment, Error bar = 1 Std. (C) Representative images of lamin A/C null CFs stained with DAPI (405nm), Phalloidin Texas-Red (Actin, 561nm), and lamin B (640nm). Quantifying the lamin B ring thickness around the nucleus showed no significant difference between TCP and ROS-induced lamin A/C null CFs. N = 7 animals/group, n ≥ 25 nuclei/treatment, Error bar = 1 Std. Scale bar = 10µm. (D) ROS-induced lamin A/C null CFs had decreased chromatin condensation compared to TCP CFs. N = 7-9 animals/group, n ≥ 25 nuclei/treatment, Error bar = 1 Std. (E) Representative images show γH2A.X (488nm) staining in TCP and ROS-induced lamin A/C null CFs. Similar to WT ROS-induced senescent CFs, no significance was observed between lamin A/C null CFs in TCP and ROS-treated groups. N = 7-8 animals/group, n ≥ 25 nuclei/treatment, Error bar = 1 Std. Scale bar = 10µm. (F) The gene expression profile of the ROS-induced lamin A/C null CFs had significantly increased p16 and p19 expression. N = 6 animals. Error bar = SEM. Linear mixed model, ANOVA. ****p <0.0001, **p<0.01, *p<0.05.

**
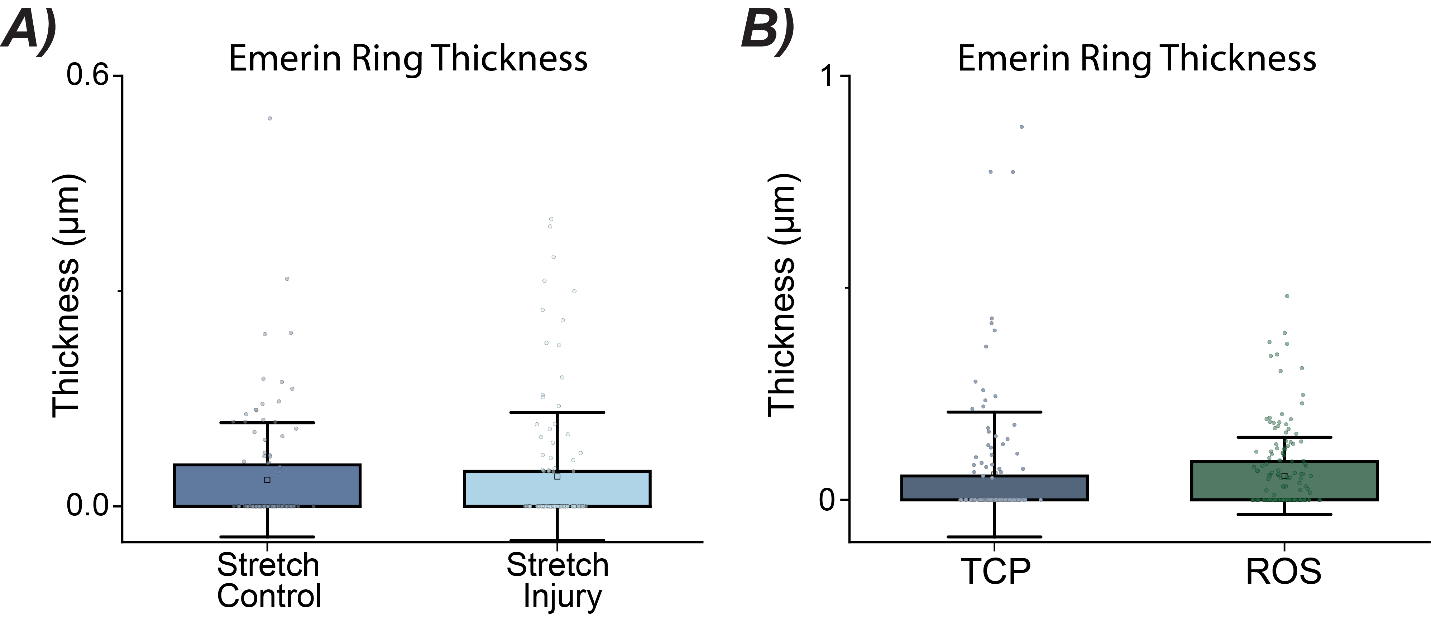
Figure S6. Emerin differences not observed in stretched and ROS-induced lamin A/C null CFs.** (A) Quantified emerin ring thickness was similar in Stretch Control and Stretch Injury lamin A/C null CFs. N = 5 animals. n ≥ 25 nuclei/treatment, Error bar = 1 Std. (B) No difference in quantified emerin ring thickness was observed in lamin A/C null CFs on TCP or treated with hydrogen peroxide (ROS-induced). N = 5-7 animals, n ≥ 25 nuclei/treatment, Error bar = 1 Std.

**Table S1: Primer sequences for qPCR of selected genes.**

| **Gene** | **Forward** | **Reverse** |
| --- | --- | --- |
| *Trp53* (*p53*) | TTCATTGGGACCATCCTGGC | GGCAGTCATCCAGTCTTCGG |
| *Mapk14* (*p38)* | AGCTGTCGAGACCGTTTCAG | GGTCACCAGGTACACGTCATT |
| *Cdkn2A* (*p16*) | GGGTTTCGCCCAACGCCCCGA | TGCAGCACCACCAGCGTGTCC |
| *Cdkn2A* (*p19*) | GGGTCGCAGGTTCTTGGTC | AATCTGCACCGTAGTTGAGCA |
| *Cdkn1a* (*p21*) | AGACATTCAGAGCCACAGGC | GACAACGGCACACTTTGCTC |
| *Gapdh* | CAAGCTCATTTCCTGGTATGAC | TGCTCAGTGTCCTTGCTGG |
